# Ellagic Acid Effect on the Components of Metabolic Syndrome, Insulin Sensitivity and Insulin Secretion: A Randomized, Double-Blind, Placebo-Controlled Clinical Trial

**DOI:** 10.3390/jcm11195741

**Published:** 2022-09-28

**Authors:** Gladys Maribel Hidalgo-Lozada, Angélica Villarruel-López, Esperanza Martínez-Abundis, Olga Vázquez-Paulino, Manuel González-Ortiz, Karina Griselda Pérez-Rubio

**Affiliations:** 1 Institute of Experimental and Clinical Therapeutics, Department of Physiology, Health Sciences University Center, University of Guadalajara, Guadalajar 44340, Mexico; 2 Department of Pharmacobiology, University Center for Exact and Engineering Sciences, University of Guadalajara, Guadalajara 44430, Mexico; 3 Health Biomedical Research Center, Guadalajara 44140, Mexico

**Keywords:** ellagic acid, insulin secretion, insulin sensitivity, metabolic syndrome, impaired fasting glucose

## Abstract

Metabolic syndrome (MetS) is a cluster of cardiovascular risk factors, usually with a common pathophysiological origin in insulin resistance and abdominal obesity. Considering the reported effects of ellagic acid (EA) on insulin resistance and abdominal obesity, the aim of this study was to evaluate the effect of EA on the components of MetS, insulin sensitivity and insulin secretion by conducting a randomized, double-blind, placebo-controlled, clinical trial with 32 volunteers diagnosed with MetS. Sixteen patients were randomly allocated, received 500 mg of EA orally twice a day for 12 weeks, and the other 16 received a placebo. Clinical and laboratory determinations were obtained at baseline and at the end of the study. After EA administration, patients reduced their waist circumference (females: 102.2 ± 4.2 to 99.5 ± 3.2 cm (*p* < 0.05); males: 99.8 ± 6.7 to 96.0 ± 4.7 cm (*p* < 0.01)), systolic blood pressure (118.1 ± 10.1 to 113.7 ± 7.8 mmHg (*p* < 0.01)), diastolic blood pressure (118.1 ± 10.1 to 113.7 ± 7.8 mmHg (*p* < 0.01)), triglycerides (2.8 ± 1.1 to 2.1 ± 0.7 mmol/L (*p* < 0.01)), fasting plasma glucose (6.5 ± 0.5 to 5.7 ± 0.6 mmol/L (*p* < 0.01)), fasting plasma insulin (*p* < 0.01), and insulin secretion (*p* < 0.05), with an increase of insulin sensitivity (*p* < 0.01). In male patients, high-density lipoprotein cholesterol increased (*p* < 0.05). In conclusion, EA improved the components of MetS, reduced hyperinsulinemia, and improved insulin sensitivity.

## 1. Introduction

Metabolic syndrome (MetS) is the coalescence of cardiovascular risk factors that include abdominal obesity, hyperglycemia, dyslipidemia, and arterial hypertension [1]. It is an important public health issue due to its high prevalence worldwide, and moreover, the individual and combined components have been associated with a significant risk of type 2 diabetes mellitus (T2DM) [1,2,3,4,5], chronic kidney disease (CKD) [2,3] and cardiovascular disease (CVD) [1,2,3,4,5]. This progression is caused by multiple factors, including glucotoxicity, lipotoxicity, chronic inflammation and increased oxidative stress [1,2,3,4,5]. These are directly related to obesity and insulin resistance, which are pathophysiological components of MetS that show a direct association with oxidative stress [5]. Oxidative stress represents a significant role in the development and progression of each MetS component and other related complications such as endothelial dysfunction, arterial stiffness, and adipose tissue dysfunction, perpetuating the cycle of multiorgan damage [1,5]. Arterial stiffness is a marker of subclinical vascular damage and a predictor for CVD in patients with MetS [5,6]. SBP and DBP are directly linked to arterial stiffness; moreover, this can be assessed through the measurement of pulse wave velocity [6]. Adipose tissue dysfunction also has an important contribution to MetS and subsequent complications [5,7]. MetS requires a comprehensive treatment, currently based on changes in lifestyle and a drug for each component [1,4]. Therefore, a treatment for all the components of MetS is necessary to avoid polypharmacy, improving the success of therapy and reducing the incidence of adverse effects [4].

Ellagic acid (EA) is a dimer of gallic acid found in pomegranates, berries, nuts, and other natural sources [8]. It has shown positive results on the isolated components of MetS in vitro and in vivo [8]. These components share pathophysiological mechanisms related to insulin resistance and abdominal obesity [1]. Among the reported effects is the reduction of insulin resistance [9,10,11] by suppressing the secretion of the adipocytokine resistin [11]. Obesity and other metabolic disorders cause an increase in this cytokine, leading to T2DM [11]. Resistin suppression by EA contributes to the prevention of T2DM [11]. Poulose et al. [12] reported that EA consumption increased the translocation of the glucose transporter 4 (GLUT-4) in skeletal muscle and adipose tissue through the activation of adenosine monophosphate-activated protein kinase (AMPK) and its downstream protein, the extracellular signal-regulated kinase (ERK) [12]. These in vitro effects explain the improvement of fasting plasma glucose (FPG) reported in human studies [9,10]. Panchal et al. [13] observed a reduction in abdominal obesity and omental fat in rats [13]. Similar outcomes from an in vitro study were reported by Wang et al. [14], who found that EA inhibited adipogenesis through the suppression of adipocytes differentiation, interrupting the cell cycle [14]. It has been observed that the consumption of EA improves plasma lipid levels through various mechanisms [8], like increasing the secretion of apolipoprotein apoA-1, the main apolipoprotein of the high-density lipoprotein cholesterol (HDL-c); and by inhibiting apolipoprotein B, a component of low-density lipoprotein cholesterol (LDL-c) and very low-density lipoprotein (VLDL) [15]. EA also upregulates the expression of the LDL-c receptor, via the ERK signaling pathway, increasing the LDL-c uptake [16]. The effect of EA on triglycerides could also be related to the suppressive effect at the genetic level of EA on microsomal triacylglycerol transfer protein [16]. EA reduces systolic blood pressure (SBP) [13], through upregulating the endothelial nitric oxide synthase [17]; this effect increases the bioavailability of nitric oxide, a decrease associated with endothelial dysfunction [18] and an increase of blood pressure [18]. According to reports, EA has shown a broad safety profile and no adverse effects [9,10,19]. 

To our knowledge, there are four published works on humans evaluating EA on some metabolic parameters [9,10,20,21]. However, it has not been directly assessed in patients with MetS. For this reason, it is necessary to evaluate the effect of EA consumption on all the components of MetS, since EA could contribute to a comprehensive treatment. The objective of this trial was to evaluate the effect of EA on all the components of MetS, insulin sensitivity, and insulin secretion in patients with diagnosed MetS. 

## 2. Materials and Methods

### 2.1. Study Design

A randomized, double-blind, placebo-controlled, clinical trial was conducted. Stages of the study included screening, basal testing, allocation, intervention, final testing, and statistical analysis.

### 2.2. Subjects

Thirty-two patients with a MetS diagnosis were included in the study. Screening and enrollment of volunteers were performed at the Institute of Experimental and Clinical Therapeutics, at the Universidad de Guadalajara, from 2019 to 2021, according to the enrollment criteria in Table 1.

### 2.3. Study Variables

The primary variables stablished were waist circumference, SBP, diastolic blood pressure (DBP), FPG, triglycerides, HDL-c, insulin sensitivity and secretion. The secondary variables for this study were: body weight, body mass index (BMI), body fat mass (BFM), glucose concentration at 120′ post-load (2h-PG), fasting plasma insulin (FPI), area under the concentration–time curve (AUC) for glucose and insulin, total cholesterol, LDL-c, VLDL and uric acid. The safety variables were also considered: aspartate aminotransferase (AST), alanine aminotransferase (ALT), creatinine, and the occurrence of adverse effects.

### 2.4. Randomization

A simple randomization by an external researcher was performed using a computer-generated randomization scheme. A numerical code was assigned to sealed intervention containers. Patients were allocated 1:1 to the EA group or the placebo group.

### 2.5. Pharmacological Intervention

**Figure 1:**
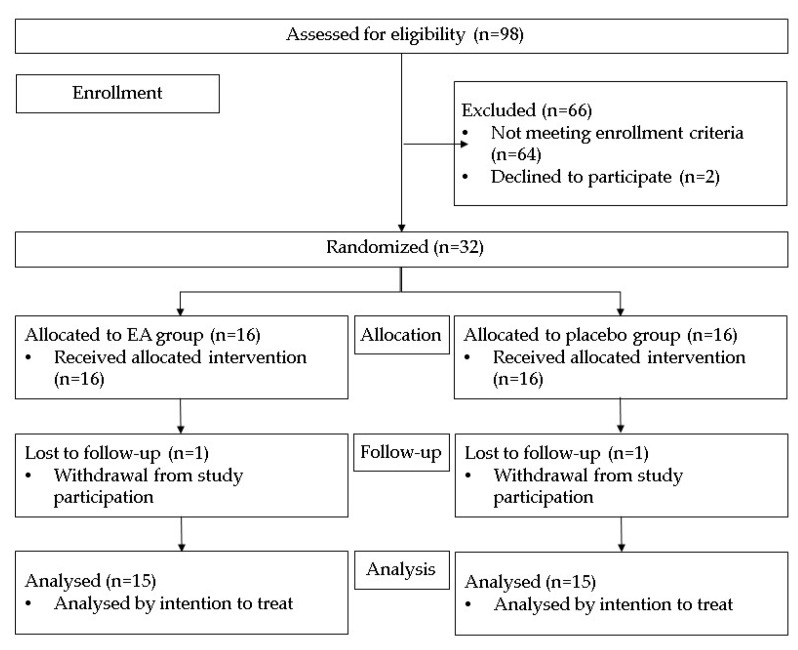
Enrollment flowchart.

A total of 32 volunteers were included in the trial and allocated into two groups (Figure 1). The treatment arm comprised 16 patients receiving EA (Pure Bulk, Inc. Pomegranate extract EA 90%), a 500 mg capsule with oral dose every 12 h for 12 weeks. The control arm consisted of 16 patients receiving a homologated placebo (calcined magnesia), a 500 mg capsule oral dose every 12 h for 12 weeks.

### 2.6. Blinding

All study participants, researchers, and the statistical analyzer were blinded for the intervention. Numbers and assignments stayed blind until the final statistical analysis was reached.

### 2.7. Procedures

Clinical and laboratory determinations were performed at 8:00 a.m., after a 12 h overnight fast. Determinations from all the participants were obtained at baseline and after 12-week intervention.

#### 2.7.1. Clinical Determinations

Waist circumference was measured with the patient standing on both feet, straight back, at the end of a normal expiration, using a flexible glass-fiber metric tape placed on a horizontal plane around the waist, at the approximate midpoint between the lower margin of the last palpable rib and the top of the iliac crest in the mid-axillary line, with snug tape, avoiding skin compression, in parallel to the floor. All waist circumference measurements were obtained by the same researcher and registered in centimeters (cm) to one decimal place.

Height, body weight, BMI, and BFM were obtained using a bioimpedance electric scale TANITA^®^ TBF215GS body mass analyzer, according to the manufacturer’s instructions. All fasted patients had minimal clothing, no metallic objects, with an empty bladder and digestive system, clean soles of their feet centered on the metallic electrodes, with a straight back and head aligned in Frankfort horizontal plane from the beginning till the end of the procedure. Height was reported in cm, body weight was reported in kilograms (kg), and BFM was reported in percentage. SBP and DBP were obtained by a digital sphygmomanometer (OMRON HEM-7130) in accordance with the American Heart Association recommendations [23]. Three measurements were obtained for each arm at 1–2 min intervals, and mean was registered reporting millimeters of mercury (mmHg). 

#### 2.7.2. Laboratory Determinations

A 2 h 75 g oral glucose tolerance test (OGTT), sampling every 30 min, was performed to measure glucose and insulin concentrations. The remaining laboratory determinations were analyzed from the fasting blood sample. Female patients’ blood samples were obtained between days 3 and 8 of their menstrual cycle (follicular phase). All blood samples were collected using an aseptic technique, from the median cubital vein through venipuncture, to vacuum-sealed sterile tubes without anticoagulant. After centrifugation at 4000 rpm for 15 min, the obtained plasma was pipette-transferred to microtubes to process chemical variables. Aliquots for insulin quantification were frozen at –20 °C and only defrosted for their analysis. Chemical determinations of glucose, triglycerides, total cholesterol, HDL-c, LDL-c, VLDL, uric acid, AST, ALT, and creatinine were performed by the dry chemistry analyzer Fujifilm DRI-CHEM NX500 with an accuracy of 0.75 g/dL, and a precision of 0.25 g/dL (intra-assay variability ≤ 5%). Insulin determinations were performed by chemiluminescent immunoassay in the IMMULITE system with an accuracy of 2 µUI/mL, and intra-assay variability of ≤5%.

#### 2.7.3. Calculations

The AUC for glucose and for insulin were estimated by the trapezoidal rule. First, phase insulin secretion was calculated with the Stumvoll index: 1283 + 1.829 × insulin 30′ − 138.7 × glucose 30′ + 3.772 × insulin 0′ [24]. Total secretion of insulin was estimated by the ratio AUC of insulin: 0–120/AUC of glucose 0–120 [25], and insulin sensitivity was calculated by the Matsuda index: 10,000/square root of (glucose 0′ × insulin 0′) × (mean glucose × mean insulin during OGTT) [26].

### 2.8. Follow-Up for Treatment Adherence 

Treatment adherence was followed through with scheduled visits every four weeks, direct interview, a treatment-log diary where patients made registers, and the counting of residual capsules to verify an intervention adherence > 80%. Patients received general recommendations of nutrition and physical activity [27]. Physical activities of all subjects were estimated around three to five metabolic equivalents (moderate physical activity) previous and during the study.

### 2.9. Ethical Considerations

The study was conducted according to the ethical standards for medical research involving humans in the Declaration of Helsinki [28], following approval by the Ethics Committee of the Health Sciences University Center at Universidad de Guadalajara (CEI/488/2019), and registration at ClinicalTrials.gov (NCT04916379).

During the follow-up, patients were encouraged to register in the treatment log diary any reaction or possible adverse effect. Each report was registered, and causality was analyzed. Adverse events were reported to the Ethics Committee.

### 2.10. Statistical Analysis

Sample size was calculated using the mean differences formula for each primary variable, with statistical power at 80% and statistical confidence at 95%. In accordance with the largest sample size obtained (triglycerides), we considered an anticipated difference of 0.20 mmol/L [29] and standard deviation (SD) of 0.18 mmol/L [30] in the MetS population. A total of 16 subjects per group was obtained, including an added 20% of anticipated losses. Statistical analysis was carried out in the IBM SPSS Statistics software version 26 for Windows. The Shapiro–Wilk test and quantile–quantile plots were carried out to evaluate data distribution. Homoscedasticity was assessed through the Levene test reporting homogeneous variances. A multiple comparison adjustment was performed to identify relevant endpoints. The intergroup differences were examined through the Mann–Whitney U test for basal data, intergroup final analysis and for mean of change differences, considering a two-tailed analysis, α = 0.05 and a 95% confidence interval (CI). The intra-group analysis was carried out through the Wilcoxon signed-rank test with the same CI. Qualitative variables were analyzed by Fisher’s exact test. Quantitative variables were reported as mean ± SD. Values were expressed in accordance with the International System of Units (SI). A *p* ≤ 0.05 was considered statistically significant. 

## 3. Results

### 3.1. Study Population

Thirty-two patients with MetS diagnosis [22] fulfilled the selection criteria and were randomly included in two groups: 16 for the EA treatment (six (38%) males and 10 (62%) females); and 16 for the placebo group (four (25%) males and 12 (75%) females). The mean age of patients was 43 ± 7 and 44 ± 9 years, for the EA and placebo groups, respectively (*p* = 0.70). During the trial, one patient from each group withdrew from the study for reasons unrelated to the intervention. Then, 15 volunteers in each group completed the intervention over 12 weeks (Figure 1) with treatment adherence of >90%. There were no significant differences between groups at baseline (*p*~0.270) (Table 2 and Table 3).

### 3.2. Clinical Results

#### 3.2.1. Primary Outcomes

The comparison between changes from baseline to week 12 for EA and placebo showed that patients in the EA group reduced their waist circumference (−2.5 ± 1.5 (95% CI: −3.3 to −1.7) vs. 0.2 ± 1.7 cm (*p* = 0.001)), SBP (−4.93 ± 6.2 (95% CI: −8.4 to −1.5) vs. −0.1 ± 2.3 mmHg (*p* = 0.011)) and DBP (−3.13 ± 3.1 (95% CI: −4.8 to −1.4) vs. −0.4 ± 2.6 mmHg (*p* = 0.013)) (Table 3). The mean difference of changes between groups was also carried out and the results are reported in Table 4.

#### 3.2.2. Secondary Outcomes

The comparison between changes in the EA group vs. the placebo group showed that patients in the EA group reduced their body weight (−1.5 ± 1.9 (95% CI: −2.6 to −0.4) compared to 0.71 ± 1.2 kg in the placebo group (*p* = 0.001)) and BMI (−0.6 ± 0.8 9 (95% CI: −1.0 to −0.1 vs. 0.3 ± 0.5 cm (*p* = 0.001)) (Table 3). The results of the mean difference of changes between groups for these variables are also reported in Table 4. The BFM determination reported no significant changes in both groups (*p* = 0.178).

### 3.3. Laboratory Results

#### 3.3.1. Primary Outcomes

Patients in the EA group showed a higher reduction in some laboratory outcomes compared to the placebo group. FPG (−0.8 ± 0.6 (95% CI: −1.1 to −0.5) vs. 0.21 ± 0.4 mmol/L (*p* = 0.001)), triglycerides (−0.7 ± 0.9 (95% CI: −1.3 to −0.2) vs. 0.5 ± 0.6 mmol/L (*p* = 0.001)), first phase of insulin secretion by Stumvoll index (−375 ± 532 (95% CI: −669 to −80) vs. 66 ± 329 pmol/L (*p* = 0.023)), and increasing of insulin sensitivity by the Matsuda index (1.4 ± 1.1 (95% CI: 0.7 to 1.9) vs. −0.2 ± 0.4 (*p* = 0.001)). Insulin secretion by ratio insulin AUC/glucose AUC showed not significant diminution (*p* = 0.091). HDL-c showed a statistically significant increment in male patients compared to the placebo group (*p* = 0.002); females showed a not-significant increase (Table 2).

#### 3.3.2. Secondary Outcomes

The comparative mean differences of changes reported a major diminution for the EA group vs. the placebo group in 2h-PG (−1.45 ± 1.7 (95% CI: −2.4 to −0.5) vs. 0.9 ± 1.8 mmol/L (*p* = 0.005)), glucose AUC (−138 ± 162 (95% CI: −228 to −48) vs. 127 ± 220 mmol/L/min (*p* = 0.001)), FPI (−61.7 ± 69.0 (95% CI: −99.9 to −23.5) vs. 12.2 ± 33.8 pmol/L (*p* = 0.001)), insulin AUC (−21,474 ± 16,548 (95% CI: −30,638 to −12,310) vs. 9917 ± 12,695 pmol/L/min (*p* = 0.001)), VLDL (−0.15 ± 0.2 (95% CI: −0.3 to −0.04) vs. 0.1 ± 0.1 mmol/L (*p* = 0.001)) and uric acid (−20.6 ± 31.2 (95% CI −37.9 to −3.3) vs. 18.6 ± 30.8 µmol/L (*p* = 0.002)) (Table 3). The secondary outcomes showed that total cholesterol and LDL-c remained with no significant changes in the intragroup and intergroup analyses (total cholesterol, *p* = 0.073; for LDL-c, *p* = 0.446).

### 3.4. Adverse Events

Through the trial, six patients reported feces softening (+1 point of Bristol stool scale), five from the placebo group (31.3%) and one from the EA group (6.3%). It was registered as an adverse event that did not require medical attention nor interrupting the intervention and was not statistically significant (*p* = 0.172). Concentrations of AST, ALT and creatinine were measured at baseline and at the end of the study. The intergroup analysis reported no statistically significant differences for changes in the following safety variables: AST, *p* = 0.464; ALT, *p* = 0.716; creatinine, *p* = 0.206.

## 4. Discussion

A MetS pharmacological comprehensive treatment is still under research [1]. Two therapeutic targets could be insulin resistance and abdominal obesity, since these are important factors identified in the pathophysiology of all the MetS components [1,5]. Insulin resistance promotes the increase of free fatty acids, with associated alterations on the insulin signaling pathway and the decrease in translocation of GLUT-4 [1]. The progression of these abnormalities leads to hyperinsulinemia in an effort of pancreatic β-cells to provide the required insulin, which is not reaching cells because of a dysfunction in the insulin receptor signaling pathway [3,31]. This compensatory insulin hypersecretion promotes abdominal obesity, increasing insulin resistance through adipocytokines release [11] and subsequent insulin hypersecretion, leading to more obesity and to each MetS component [1,5]. EA effects on insulin resistance [9,10,11] suggest a potential effect on mechanisms and components of MetS. However, only four studies in humans about the effect of ellagic acid on metabolic parameters have been published [9,10,20,21]. 

### 4.1. Abdominal Obesity

This is the first study to evaluate the effect of EA on patients with MetS. The main criterion was abdominal obesity [22]. A limitation of this study was the omitted measurement of this with the gold standard computed tomography scanning; however, the waist circumference was used, which is a diagnosis criterion for MetS [20] and exerts a correlation of 0.74 for abdominal fat [32]. Another obtained variable was the BMI, which correlates at 0.82 to abdominal fat [32]. Both waist circumference and BMI showed a statistically significant diminution in the EA group, suggesting a diminution of abdominal obesity. Liu et al. [20] conducted a study with healthy normal-weight men and overweight men with dyslipidemia; after an AE intervention for 12 weeks, participants reduced their waist circumference and BMI [20]. A possible mechanism could be the reduction of adipogenesis through EA inhibition in adipocytes differentiation [14]. Despite the reported reductions in waist circumference, BMI, and body weight in patients from the EA group in this study, total BFM showed no significant differences. This is similar to the outcomes of a study with rats where, after EA administration, it was reported a diminution of abdominal fat, but not a significant decrease of total body fat [13]. Redistribution of body fat promoted by EA is possible considering that it suppresses adipogenesis and insulin resistance [14], while promoting the intake of free fatty acids and LDL-c in the adipose tissue [16]. Notwithstanding the absence of a BFM reduction due to MetS pathophysiology, the potential reduction of abdominal obesity by EA might improve other components of MetS.

### 4.2. Arterial Hypertension

Abdominal obesity and hyperinsulinemia promote increased oxidative stress and dysfunction of the renin–angiotensin–aldosterone system and the activation of intrarenal angiotensin II [3,5,33]. The combined metabolic impairments produce progressive endothelial dysfunction, vascular stiffness, and neuroimmune alterations that promote low nitric oxide bioavailability even in normotensive patients with predisposition to develop hypertension [5,33]. This pre-hypertensive status could be prevailing in patients with MetS. In our study, the EA group showed a significant reduction of SBP and DBP. Variability in results with EA can be justified by the multifactorial pathophysiology of hypertension and the fact that EA has only been proven based on SBP reduction by upregulating nitric oxide bioavailability in rats [17]. Besides in our outcomes, EA showed no effect on patients with SBP under 110 mmHg or with SBP under 68 mmHg. This is in accordance with Berkban et al., who reported a reduction of blood pressure in hypertensive subjects, and no effects in normotensive ones [17]. This is relevant since it has been reported that patients with high-normal blood pressure (130–139/80–89 mmHg) require pharmacological support, additional to dietitian therapy to achieve control goals [34]. However, most of them do not qualify for immediate drug therapy [34]. This, due to hypotension and other adverse effects related to medication, is common in patients with normal blood pressure under pharmacological treatment [35]. To our knowledge, this is the first clinical trial assessing the effects of EA on SBP and DBP. A limitation of this study is the absence of assessment of stress/inflammation markers, arterial stiffness or endothelial dysfunction More studies are necessary to identify whether EA could be a resource to treat patients with “high-normal blood pressure” or arterial hypertension. This is a complementary therapy in addition to a lifestyle adjustment. Moreover, long-term cohort studies could provide information about EA as a hypertension-prevention tool for high-risk patients. 

### 4.3. Plasma Glucose

Insulin resistance and abdominal obesity produce direct effects on glucose metabolism [1]. Dysregulations between glucose absorption, cellular intake, storage, and synthesis promote high concentrations of fasting and postprandial glucose. Insulin resistance and subsequent hyperinsulinemia add a dysfunction of the insulin effect to suppress the hepatic glucose production; this through glycogen degradation (glycogenolysis) or de novo synthesis of glucose (gluconeogenesis) [36,37]. EA exerts an effect as a competitive inhibitor of glycogen phosphorylase enzyme to avoid glycogenolysis [38]. In this study, to obtain FPG and emulate the postprandial increase of glucose, a 2 h 75 g OGTT was carried out. Our findings are concordant with the study of Ghadimi et al., where patients with T2DM threated with metformin received EA as a complementary therapy, and a documented reduction of FPG and 2h-PG [9]. Similar results were reported by Kazemi et al. with a reduction of FPG after EA administration to female patients with metformin-treated insulin resistance [10]. These modifications could be related to EA regulation of endogenous glucose production and glucose metabolism [25,26,27,28,29,30,31,32,33,34,35,36,37,38]; however, other measurements such as glucagon, which is regulated by glucose and suppressed by hyperinsulinemia [36], could be useful to a wide pathophysiological comprehension. 

Besides, no episodes of hypoglycemia have been reported during in vivo studies, neither prior nor during this clinical trial. It is especially remarkable considering that patients with impaired glucose or insulin resistance need available therapies without the risk of hypoglycemia. EA could be a complementary therapy for patients with impaired fasting glucose, postprandial hyperglycemia with normal FPG, or related alterations with a risk of hypoglycemia and insufficient control with only lifestyle changes. However, other phase II and III studies are necessary to assess EA on this populations.

### 4.4. Insulin Sensitivity and Secretion

The insulin-signaling pathway is impaired in MetS due to multiple factors, highlighting abdominal obesity and insulin resistance [1]. This insulin-impaired sensitivity promotes an increased secretion of insulin, creating a vicious cycle since a hyperinsulinemia status leads to more insulin resistance [1,3,31]. This study assessed insulin secretion and insulin sensitivity. Multiple formulas to calculate insulin secretion are available [39]. In this study, first-phase insulin secretion was estimated through the Stumvoll index based on insulin levels at 0 and 30 min in an OGTT, since this formula correlates well (0.75) with the gold standard for insulin secretion [24]. An estimation of total insulin secretion, which is interpreted as pancreatic β-cell response, was obtained by the ratio AUC of insulin/AUC of glucose considering 0 and 120 min for both values [39]. Insulin sensitivity was analyzed using the Matsuda Index, since it provides a correlation ≥ 0.73 with the gold-standard test and the hyperinsulinemic euglycemic clamp [26]. Our findings are concordant with the studies of Ghadimi et al. and Kazemi et al.; they reported a reduction of FPI, and insulin resistance [9,10]. These changes could be promoted by EA through enhancing GLUT-4 translocation [12] in addition to its effect to suppress the secretion of the adipocytokine resistin. Resistin is released as part of the adipose dysfunction to produce higher insulin resistance [11]; however, this study was not guided to measure adipocytokines nor the molecular expression of glucose transporters, so resistin and GLUT-4 translocation were not assessed. Perhaps these effects could explain the improvement of hyperglycemia, hyperinsulinemia, and insulin sensitivity in the EA group. Hyperinsulinemia improvement could also be related to the enhanced insulin sensitivity, through the interruption of the compensatory response [31].

### 4.5. Triglicerides

Abdominal obesity and, particularly, visceral fat, have been studied as the main participants in dyslipidemia due to adipocytokines and free fatty acids release [11]. Hypertriglyceridemia is a high-prevalence disorder [40], marker and component of MetS [22]. Triglycerides in healthy subjects must be stored in adipocytes; nevertheless, in insulin resistance, the desensitized adipose tissue cannot suppress lipolysis, releasing more free fatty acids and contributing to VLDL formation and hypertriglyceridemia [41]. Therefore, the decrease of triglycerides and VLDL could be explained with the EA effect improving insulin sensitivity. A limitation of this trial is the absence of strict control of dietary intake; however, our findings are according to reports of previous studies [10,20]. Although most cardiovascular associations and guides do not consider necessary the treatment of triglycerides <5.7 mmol/L [42], considering the adverse effect of hypertriglyceridemia on insulin sensitivity and β-cell function [40], it must be necessary to take care of triglycerides control since the initial increase. EA could be a complementary therapy for high triglycerides dyslipidemia mainly for subjects with fibrates intolerance; however, more studies are necessary. 

### 4.6. HDL-c

Reports about the EA effect on HDL-c have been inconsistent. In a study conducted on overweight men with dyslipidemia, reductions on plasma triglycerides, VLDL, total cholesterol, LDL-c, and increment of HDL-c after EA administration for 12 weeks were reported [20]. Besides, Kazemi et al. evaluated metformin vs. EA + metformin in women, and Ghadimi et al. assessed metformin compared to EA + metformin in both sexes [9,10], reporting diminution of triglycerides, total cholesterol and LDL-c, but not a significant increase of HDL-c. In our study, females showed a not-significant HDL-c increase, and males reported a significant increment of HDL-c. Differences between studies can be related to diet and physical activity or individual characteristics; however, the main difference might be the sex. Liu et al. assessed men with a statistically significant HDL-c increase [20], Kazemi and coworkers studied a population of women and reported no increase of HDL-c [10], Ghadimi et al. studied both sex patients, but analyzed outcomes without sex separation [9]. In our study, statistical analysis of HDL-c was carried out with sex separation.

The mentioned trials showed reductions on total cholesterol and LDL-c. It has been reported the effect of EA upregulating the expression of the LDL-c receptor increasing the LDL-c uptake [16], and its effect increasing the secretion of the apolipoprotein apoA-1 (major apolipoprotein HDL-c), inhibition of apolipoprotein B (component of LDL-c and VLDL) [15], and gen-level suppressor action of EA on the microsomal triacylglycerol transfer protein [16] that mobilizes triglycerides to ApoB. Despite these reported effects, in our study, total cholesterol and LDL-c showed no significant reductions. Differences between prior trials and our study must be related to metformin administration, which could produce an additive or synergic effect [43]. In our study, patients did not receive additional drugs, products, or supplements.

### 4.7. Uric Acid

Several studies have reported a positive statistical correlation between insulin resistance and plasma uric acid [2]. Hyperuricemia exerts positive association with obesity, hypertension, MetS, and cardiometabolic complications [44,45]. Hyperuricemia is also a risk predictor for hypertension, CVD, T2DM and CKD [2,5,45], sharing as a pathological mechanism the uric acid inhibition of endothelial nitric oxide bioavailability [2,5]. It has been reported an effect of EA upregulating endothelial nitric oxide synthase and restoring nitric oxide bioavailability [17]. Another study reported a direct effect of EA, reducing uric acid synthesis by inhibition of xanthine oxidase [46]. This inhibition reduces tissue injury, target-organ damage, and cardiovascular risks [46]. An important related fact is that in a long-term cohort for mortality analysis, it was identified that uric acid > 5.1 mg/dL (303 µmol/L) in females, or >5.6 mg/dL (333 µmol/L) in males, can be a predictor of cardiovascular mortality with linear association between serum uric acid and increased risk of all-cause mortality and cardiovascular mortality [47]. These reports show a new group of patients who need a treatment and could benefit from EA therapy starting from the initial elevation of uric acid, when drugs have no indication yet. More studies in humans are necessary to identify whether EA could be a choice to treat hyperuricemia considering that high uric acid in plasma is an independent risk factor for CVD and metabolic diseases, but current treatments have no indication for asymptomatic hyperuricemia [45]. To our knowledge, there are no prior clinical studies evaluating the EA effect on uric acid. In this study, it was found out that EA reduces uric acid concentrations.

In our results, the increase of insulin AUC, triglycerides and VLDL for the baseline–end intragroup analysis for placebo group patients are expected changes since insulin resistance is directly associated to insulin secretion as well as to triglycerides and VLDL concentrations [1,3,31,41].

Participants in this study were general city-population, diagnosed in our screening without previous intervention. In this study, we selected the International Diabetes Federation criteria for MetS diagnosis [22] due to its inclusion of abdominal obesity as a main criterion, matches with MetS pathophysiology [1]. Moreover, its adjustment of waist circumference in accordance with the population improves the diagnostic certainty [22]. Patients included in this study match with the harmonized criteria for MetS [48] and most of the proposed diagnosis criteria [2]. Safety limits were stablished as exclusion criteria. The applied dose of EA for this essay was the suggested by the supplier due to a protocol designing date, there were no clinical studies reporting the effects of EA on glucose and metabolic parameters. The not-adverse effect level for EA was respected [19]. EA is possibly a wide safety margin molecule, and no significant adverse effects were identified in this trial. 

This study’s outcomes offer knowledge about the effect of EA on patients with MetS. This is relevant because most information proceeds from in vitro and in vivo studies which are not always concordant with studies in humans. Additionally, this trial offers information about high-dose EA effects. New assays can be conducted to identify the human minimum effective dose for each component. EA must be studied in a phase 3 clinical trial for inferential information, as single or complementary therapy in some component of MetS or other related disease, offering to patients the benefit of multiple effects through the same regulation pathway.

## 5. Conclusions

Ellagic acid consumption after 12 weeks of enhanced insulin sensitivity decreased insulin secretion and improved MetS components. It reduced abdominal obesity, blood pressure, fasting glucose, and triglycerides for males and females with an increasing of HDL-c in only male patients. Additionally, ellagic acid decreased uric acid, VLDL and AST.

## Figures and Tables

**Table 1:** Inclusion, exclusion, and elimination criteria.

Selection Criteria
**Inclusion criteria** Male and female 30 to 59 years old Diagnosis of MetS according to the International Diabetes Federation criteria [22] Signed informed consent
**Exclusion criteria** Prior diagnosis of liver, kidney, pancreas, heart, or thyroid disease T2DM or arterial hypertension History of alcoholism, drug abuse or tobacco use SBP ≥1 40 mmHg and/or DBP ≥90 mmHg Body mass index ≥35.0 kg/m^2^ Triglycerides ≥ 5.7 mmol/L FPG ≥ 7.0 mmol/L LDL-c > 4.9 mmol/L Body weight variability ≥ 5% throughout the prior last three months Suspected or confirmed pregnancy Lactation Known allergy to any of the interventions Impossibility to swallow capsules Hormonal contraceptive or replacement therapy Physical activity < 3 or >5 metabolic equivalents Pharmacological, dietary, or herbal therapy in the last three months before the trial.
**Elimination criteria** Withdrawal of informed consent Intolerance to EA or placebo Loss of follow-up Adherence to treatment < 80%

MetS, Metabolic syndrome; T2DM, type 2 diabetes mellitus; SBP, systolic blood pressure; DBP, diastolic blood pressure; FPG, fasting plasma glucose; LDL-c, low-density lipoprotein cholesterol; EA, ellagic acid.

**Table 2:** Primary outcomes at baseline and at the end of the intervention.

		Placebo				Ellagic Acid			
Baseline		*n* = 16	Week 12	*n* = 15	Baseline,	*n* = 16	Week 12	*n* = 15	*p* ***
Mean		(SD)	Mean	(SD)	Mean	(SD)	Mean	(SD)	
**MetS diagnosis criteria**									
WC, cm	♂101.4	(1.1)	♂ 101.8	(1.2)	♂102.2	(4.2)	♂99.5	(3.2) *	0.010
	♀ 95.5	(10.1)	♀ 96.70	(10.7)	♀ 99.8	(6.7)	♀ 96.0	(4.7) **	0.011
SBP, mmHg	112.0	(10.0)	112.5	(6.8)	118.1	(10.5)	113.7	(7.8) **	0.011
DBP, mmHg	74.0	(5.6)	74.3	(5.5)	77.8	(7.8)	75.2	(5.8) **	0.013
FPG, mmol/L	6.2	(0.6)	6.4	(0.6)	6.5	(0.5)	5.7	(0.6) **	0.001
TG, mmol/L	2.7	(0.8)	3.1	(1.1) *	2.8	(1.1)	2.1	(0.7) **	0.001
HDL-c, mmol/L	♂ 0.7	(0.1)	♂ 0.7	(0.1)	♂ 0.7	(0.2)	♂ 0.8	(0.2) *	0.019
	♀ 0.8	(0.2)	♀ 0.8	(0.2)	♀ 0.8	(0.2)	♀ 0.9	(0.2)	0.175
**Insulin sensitivity**									
Matsuda index	1.8	(0.4)	1.6	(0.5)	1.7	(0.8)	3.1	(1.7) **	0.001
**Insulin secretion**									
1p Stumvoll index	1494	(679)	1568	(831)	1573	(714)	1191	(614) *	0.011
Ratio AUC	72	(31)	73	(31)	69	(31)	58	(32) *	0.091

* *p* ≤ 0.05, ** *p* ≤ 0.01. Intragroup differences between baseline and week 12, analyzed by Wilcoxon signed-rank test. *p* *** Intergroup comparison. Mann–Whitney U test for mean differences. Confidence Interval (CI) of 95%. Data are expressed as mean and standard deviation (SD). WC, waist circumference; SBP, systolic blood pressure; DBP, diastolic blood pressure; FPG, fasting plasma glucose; TG, triglycerides; HDL-c, high-density lipoprotein cholesterol; 1p, first phase; ♂, male; ♀, female.

**Table 3:** Secondary outcomes at baseline and at the end of the intervention.

		Placebo				Ellagic Acid			
Baseline		*n* = 16	12-w	*n* = 15	Baseline	*n* = 16	12-w	*n* = 15	
Mean		(SD)	Mean	(SD)	Mean	(SD)	Mean	(SD)	*p* ***
**Clinical outcomes**									
BW, kg	77.6	(3.4)	78.5	(3.8)	79.9	(7.1)	78.6	(6.6) *	0.001
BMI, kg/m^2^	30.8	(1.6)	31.2	(2.0)	30.6	(2.6)	30.1	(2.8) *	0.001
**Laboratory outcomes**									
2h-PG, mmol/L	8.8	(1.8)	9.7	(1.8)	9.4	(1.6)	7.9	(1.6) **	0.001
Glucose AUC, mmol/L/min	1150	(198)	1278	(176) *	1219	(195)	1089	(159) **	0.001
FPI, pmol/L	133.4	(38.4)	142.7	(50.8)	153.1	(66.7)	93.3	(43.0) **	0.001
Insulin AUC pmol/L/min	80593	(32748)	83871	(41430) *	92678	(38379)	63447	(37072) **	0.001
TC, mmol/L	5.0	(0.9)	5.1	(0.9)	5.1	(1.0)	4.7	(1.0)	0.073
Uric acid, µmol/L	336.1	(66.3)	348.9	(63.7)	357.3	(68.4)	344.2	(53.1) *	0.002

* *p* ≤ 0.05, ** *p* ≤ 0.01. Intragroup differences between baseline and week 12, analyzed using the Wilcoxon signed-rank test. 95% CI. *p* *** Intergroup comparison. Mann–Whitney U test for mean differences. BW, body weight; BMI, body mass index; 2h-PG, 2 h post-load glucose; AUC, area under the curve; FPI, fasting plasma insulin; TC, total cholesterol. Data expressed as mean and standard deviation.

**Table 4:** Intergroup comparison at the end of the intervention. Difference in means of changes.

	Placebo Group	EA Group	Mean Difference	95% CI		*p*
				Lower	Upper	
**MetS diagnosis criteria**						
WC, cm	0.200	−2.533	2.733	1.537	3.930	0.010
SBP, mmHg	−0.067	−4.933	4.867	1.256	8.478	0.011
DBP, mmHg	−0.400	−3.133	2.733	0.611	4.855	0.013
FPG, mmol/L	0.212	−0.792	1.004	0.615	1.393	0.001
TG, mmol/L	0.480	−0.733	1.213	0.608	1.818	0.001
HDL-c, mmol/L	−0.066	0.054	−0.120	−0.190	−0.049	0.015
**Insulin sensitivity**						
Matsuda index	−0.213	1.354	−1.566	−2.201	−0.932	0.001
**Insulin secretion**						
1p Stumvoll index	66.005	−375.195	441.200	107.327	775.072	0.011
Ratio AUC	−1.094	−11.539	10.444	−1.798	22.687	0.091

Confidence Interval (CI) of 95%. WC, waist circumference; SBP, systolic blood pressure; DBP, diastolic blood pressure; FPG, fasting plasma glucose; TG, triglycerides; HDL-c, high-density lipoprotein cholesterol; 1p, first phase.

## Data Availability

All data presented in this paper are available on request to the corresponding authors and authorization of all parties involved in the study.
